# Cognitive behavioural therapy for anxiety in children and young people on the autism spectrum: a systematic review and meta-analysis

**DOI:** 10.1186/s40359-021-00658-8

**Published:** 2021-10-01

**Authors:** Shivani Sharma, Abigail Hucker, Terry Matthews, Dominique Grohmann, Keith R. Laws

**Affiliations:** School of Life and Medical Sciences, University of Hertfordshire, Hatfield, AL10 9AB UK

**Keywords:** Autism spectrum, Cognitive behavioural therapy, Anxiety, Follow-up, Self ratings, Parent ratings, Clinician ratings, Informant inconsistency

## Abstract

**Background:**

Anxiety is common in youth on the autism spectrum and cognitive behavioural therapy (CBT) has been adapted to address associated symptoms. The aim of the current systematic review and meta-analysis was to examine the efficacy of CBT for reducing anxiety in autistic youth.

**Method:**

Searches of PubMed and Scopus databases were undertaken from January 1990 until December 2020. Studies were included if they consisted of randomised controlled trials (RCTs) using CBT to reduce anxiety in autistic youth. Separate random effects meta-analyses assessed anxiety ratings according to informant (clinician; parent; child), both at end-of-trial and at follow-up.

**Results:**

A total of 19 RCTs met our inclusion criteria (833 participants: CBT N = 487; controls N = 346). Random effects meta-analyses revealed a large effect size for clinician rated symptoms (*g* = 0.88, 95% CI 0.55, 1.12, k = 11), while those for both parent (*g* = 0.40, 95% CI 0.24, 0.56; k = 18) and child-reported anxiety (*g* = 0.25, 95% CI 0.06, 0.43; k = 13) were smaller, but significant. These benefits were not however maintained at follow-up. Moderator analyses showed that CBT was more efficacious for younger children (for clinician and parent ratings) and when delivered as individual therapy (for clinician ratings). Using the Cochrane Risk of Bias 2 tool, we found concerns about reporting bias across most trials.

**Conclusions:**

The efficacy of CBT for anxiety in autistic youth was supported in the immediate intervention period. However, substantial inconsistency emerged in the magnitude of benefit depending upon who was rating symptoms (clinician, parent or child). Follow-up analyses failed to reveal sustained benefits, though few studies have included this data. It will be important for future trials to address robustness of treatment gains overtime and to further explore inconsistency in efficacy by informant. We also recommend pre-registration of methods by trialists to address concerns with reporting bias.

**Supplementary Information:**

The online version contains supplementary material available at 10.1186/s40359-021-00658-8.

## Background

Autism Spectrum Disorder (ASD) is a lifelong, neurodevelopmental condition characterised by difficulties in social interaction, social communication and restricted and/or repetitive patterns of behaviour [1]. As many as 70% of autistic people have at least one co-occurring mental health condition [2]. Studies consistently indicate a disproportionate risk of anxiety and anxiety-disorders in particular, though prevalence estimates vary considerably [2–4]. In their meta-analysis, Van Steensel et al. [3] reported a pooled prevalence of 39.6% of autistic youth aged under 18 years meeting criteria for at least one anxiety disorder aligned to the Diagnostic and Statistical Manual of Mental Health Disorders (DSM-IV) [5]. White et al. [4] propose that between 11 and 84% of autistic youth experience symptoms that have some degree of impact on functioning. Looking at all age groups, Lai et al. [2] suggest that 20% of people on the autism spectrum meet criteria for a co-occurring anxiety disorder. Variability in estimates are likely to reflect heterogeneity in study design and study quality [6–8]. Nevertheless, available evidence emphasises that anxiety disorders are far more common in autistic people than in the general population [9, 10]. This highlights the need for effective assessment and targeted intervention as part of routine clinical care.

Cognitive behavioural therapy (CBT) has been advanced as an effective, evidence-based intervention for anxiety [11–13]. This includes when delivered in adapted formats such as briefer treatment protocols [14] and e-health [15, 16]. In the United Kingdom (UK), the National Institute for Health and Care Excellence (NICE) endorses CBT as the first line of treatment for various mental health conditions, including anxiety in youth [17]. CBT is a short-term, talk based therapy that focuses on the interconnection of thoughts, feelings and behaviour. The core components of treatment protocols include psychoeducation, cognitive restructuring (i.e. identifying and challenging maladaptive thinking), mindfulness techniques, and graded exposure [18]. Across general populations, the efficacy of CBT for reducing symptoms of anxiety has been demonstrated; and includes the maintenance of treatment gains for up to 12 months amongst youth receiving CBT for anxiety disorders [19, 20].

The past decade has seen significant interest in the adaption of CBT for autistic children and adolescents. Intuitively, several factors might suggest that CBT may not be suited to this context. For example, treatment protocols often require consideration of multiple possibilities such as different causes or outcomes in situations. Restricted behavioural and cognitive profiles associated with ASD might therefore be expected to interfere with treatment engagement [22]. CBT also requires individuals to be able to attend to internal signals such as sensations and emotions. Both interoceptive [23] and introspective [24] abilities are impacted in ASD and may further compromise treatment efficacy. Withstanding such challenges, it has been argued that with suitable adaptation, CBT may work well for autistic youth. Spain and Happé [25] summarise that the structured nature of CBT, in-depth and collaborative focus on issues, alongside attention to graded exposure to practice application of skills are advantageous.

An emerging body of evidence supports the use of adapted CBT protocols for autistic youth [26–31], including across individual [26, 27], group [29, 30], parent-mediated [31], as well as online modalities [28]. Lang et al. [32] described important modifications to treatment protocols. They state that adaptation is less contingent on abilities such as introspection and emphasise the need for a more practical focus that prioritises behavioural over cognitive mechanisms for change. More recently, drawing on the views of practitioners and trialists, modifications were categorised under three areas: additions to treatment protocols to better accommodate the needs of autistic people (e.g. allowing clients not to make eye contact), leaving things out, (e.g. focus on core beliefs) and modifying conventional practice (e.g. diversifying communication techniques) [25]. With such adaptions, autistic youth can be supported in developing a good therapeutic alliance, which is important to facilitate behavioural change [33].

In a recent meta-analytic review, Perihan et al. [34] concluded that CBT has a moderate effect (*g* = − 0.66) on the reduction of anxiety in autistic youth aged under 18 years. The researchers also highlighted important moderators of treatment gains. Specifically, parent involvement and longer treatment protocols were found to advance more benefit. Although Perihan et al. is the most current evidence synthesis, its conclusions are limited by the inclusion of 7 out of 24 samples (30%) being from non-randomised studies. Whilst it is useful to summarise all available evidence, in meta-analysis, this requires consideration of study design features and adjustment of estimates of effect sizes [35]. Further, the pooling of both between-group comparisons of CBT versus controls with pre-post effect sizes within CBT groups and the pooling of anxiety measures within trials (i.e. where multiple informant measures were taken) is also problematic. An earlier meta-analysis, Ung et al. [36] reported a mean effect size of 0.71 although this reduced to 0.47 with the exclusion of one extreme outlier study. More crucially, like Perihan et al., Ung et al. also included non-randomised samples (2/14 studies) and pooled data across informants (self, parent and clinician). This could mask important discrepancies that impact the overall judgement of clinical efficacy. It is recognised that children and young people frequently differ in reporting symptom experience as compared to parents and clinicians [37].

The importance of analysing informants separately was highlighted by the meta-analysis of Sukhodolsky et al. [38]. They analysed raters separately, with effect sizes of 1.21, 1.19 and 0.68 for clinicians (k = 5), parents (k = 6) and child (k = 5) respectively. These effect sizes were reduced with the removal of one extreme outlier in each analysis to 0.89, 0.57 and 0.17 respectively. While the data from Sukhodolsky and colleagues points to some large discrepancies, the number of trials included was small. It is therefore unclear from existing evidence syntheses whether or not the informant impacts perceptions of CBT efficacy. Previous evidence reviews [34, 36, 38] have also not attended to whether the impact of CBT is sustained at follow-up, an important limitation of research into therapeutic benefit more generally [21]. To overcome these issues, the current study aimed to systematically review and meta-analyse the published literature on CBT in ASD derived from RCT’s assessing different informant ratings (clinician, parent and child) of anxiety symptoms both at end of trial and at follow-up.

## Methods

### Search strategy

This review adhered to the Preferred Reporting Items for Systematic Reviews and Meta-Analyses (PRISMA) recommendations [39] (see Additional File 1). Searches were conducted using the electronic databases Scopus and Pubmed for relevant trials using the following combination of search terms: (asperger OR autism OR “pervasive developmental disorder”) AND (anxiety OR anxi*) AND (“cognitive behavio*” OR CBT). Titles, abstracts and keywords were searched in Scopus, and all fields were searched in Pubmed. Lateral search techniques were used such as hand searching the references of papers for related articles and using Google Scholar to carryout key word searches. All databases were searched for all years from January 1990 to December 2020. The review was not pre-registered.

### Selection of studies

One reviewer (TM) screened the titles and abstracts of all retrieved studies to determine suitability for inclusion in the review using the following criteria: (1) participants were aged up to 18 years, (2) participants had a diagnosis of ASD according to DSM or ICD criteria, (3) participants were diagnosed with co-occurring anxiety disorder, (4) study was an RCT design, (5) intervention programme was based on CBT intended for anxiety, (6) primary outcome was a standardised measure of anxiety for which baseline and post treatment scores were reported (and follow-up where available), (7) study was published in a peer reviewed journal, and (8) article was published in the English language. The full texts of all eligible studies were screened by two reviewers (TM and AH). A random sample of 10% of excluded records were also checked (SS).

### Data extraction

Excel-based data extraction tables were used to record information for each study in the meta-analyses. This template included: sample size, number of participants in each group, type of control condition (e.g. waitlist, treatment as usual), co-morbid diagnoses, study design, setting, blind rating of symptoms and measure of anxiety. Participant characteristics extracted included mean age, gender distribution and diagnosis. For treatment characteristics, the CBT intervention programme, methods of therapy used (e.g. group, individual, parent involvement) and treatment duration were extracted. Data were extracted by TM and checked by AH—where any discrepancies occurred, these were checked and discussed by the whole research team.

### Statistical analysis

The effect size employed was Hedges *g,* which is the standardised difference between means, corrected for the tendency towards overestimation in small studies. Separate analyses were conducted for parent, clinician and child reported anxiety measures. All data analysis was conducted using Comprehensive Meta-Analysis Version 2.0 (http://www.meta-analysis.com/) using random effects models. Effect sizes were calculated using the post treatment scores derived separately (where possible) for anxiety ratings given by: parents, clinicians and the children themselves. Effect sizes were described using Cohen’s convention, where an effect size of 0.20 is considered small, 0.50 is moderate, and 0.80 is large. Publication bias was assessed by examining funnel plots, using the Duvall and Tweedie Trim and Fill method [40].

In cases where trials had multiple intervention groups and only one control group [41, 42], we followed Cochrane handbook guidance [43], and halved the control sample size when calculating effect sizes (to avoid double counting and thus influencing their weighting in analyses). If studies involved resampling or enlarging of samples in earlier studies, we would extract data from the largest study only (though this situation did not arise).

Statistical heterogeneity was assessed using the I^2^ test, with interpretation as follows: 0–40% as might not be important; 30–60% as may represent moderate heterogeneity; 50–90% may represent substantial heterogeneity; 75–100% representing considerable heterogeneity. Each study was assessed for risk of bias using the Cochrane risk of bias tool version 2.0 (RoB2) [44]. The RoB2 assesses bias that may arise across five domains: bias from randomisation, deviations from intended interventions, missing outcome data, outcome measurement and bias in selection of the reported results.

Although no definitive minimum number of studies is required for meta-regression, we follow the Cochrane Handbook recommendation of 10 studies for a continuous variable [43]; and for a categorical subgroup variable, a minimum of 4 studies per group [45].

Moderator analyses were conducted to investigate the impact of key trial and participant variables on outcome effect sizes, while continuous variables were assessed using meta-regression. Moderators were selected based on attributes that were deemed as important contributors to treatment efficacy, for example, treatment duration [34].

## Results

**Fig. 1:**
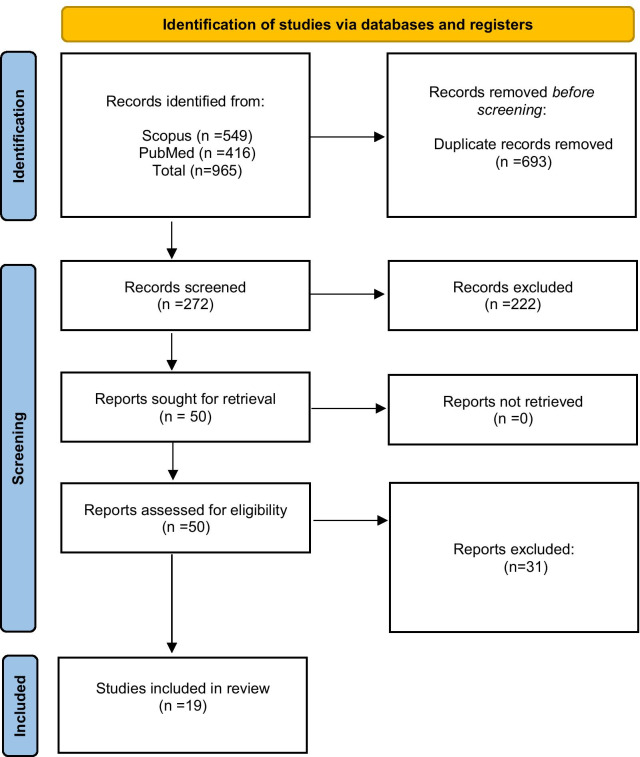
PRISMA flow diagram

A total of 965 studies were identified from the searches, of which 19 RCTs were eligible for inclusion in the meta-analysis (see Fig. 1). All studies compared the CBT intervention group to a control group (wait list control, treatment as usual or active control e.g. counselling).

### Narrative synthesis

A summary of study characteristics is provided in Table 1. Nineteen studies were included with data from 833 participants (total intervention group participants N = 487; total control group participants N = 346). Sample sizes for intervention groups ranged from 12 to 66 and for control groups ranged from 10 to 31 participants across studies. None of the 19 studies reported on adverse events or harms.

**Table 1:** Summary of included studies

Study authors	Sample size	Age M (SD)	% males	CBT type	Intervention delivery	Number of sessions	Treatment duration	Duration of follow-up	Anxiety measure	Rater
Sofronoff et al. [41]	Intervention 1: N = 23; intervention 2: N = 25; control (wait list): N = 23	Intervention 1–10.56 (0.99); intervention 2–10.54 (1.26); control—10.75 (1.04)	Intervention 1–87%; intervention 2–88%; control— 87%	CBT	Group	6	6 weeks	6 week follow-up	SCAS-P	Parent
Chalfant et al. [46]	Intervention: N = 28; control (wait list): N = 19	Overall sample—10.80 (1.35)	Overall sample—74%	Cool Kids program	Group	12	9 weekly sessions and 3 monthly booster sessions (6 months)	None reported	SCAS-P, SCAS-C, RMAS-C	Parent, child
Wood et al. [47]	Intervention: N = 14; control (wait list)l: N = 22	Overall sample—9.20 (1.49); intervention—9.18 (1.42); control—9.22 (1.57)	Intervention—71%; control—65%	Building confidence CBT program	Individual	16	16 weeks	None reported	ADIS-CSR, MASC-P, MASC-C	Clinician, parent, child
Sung et al. [48]	Intervention: N = 33; Control (Social recreational program): N = 31	Intervention—11.33 (2.03); control—11.09 (1.53)	Overall sample—94%; intervention—94%; control—94%	Modifications and adaptations were made from various CBT programs, including Coping Cat program, Exploring feelings and unpublished anxiety management programs from the Child Guidance Clinic and Autism Resource Centre	Group	16	16 weeks	3 and 6 months follow-up	SCAS-C	Child
Reaven et al. [30]	Intervention (TAU): N = 20; control: N = 23	Intervention—125.75 months (21.47); control—125 months (20.45)	Intervention—100%; control—92.3%	Facing your fears (FYF)	Group	12	12–16 weeks	None reported	ADIS-P	Clinician
McNally Keehn et al. [49]	Intervention: N = 12; control (wait list): N = 10	Overall sample—11.26 (1.53); intervention—11.65 (1.41); control—11.02 (1.69)	Intervention—100%; control—90%	Coping Cat program	Individual and two parent only sessions	16	16 weeks	2 month follow-up (CBT group only)	ADIS-P, SCAS-P, SCAS-C	Clinician, parent, child
Storch et al. [27]	Intervention: N = 22; control (TAU): N = 21	Overall sample—8.89 (1.34); intervention—8.83 (1.31); control—8.95 (1.40)	Intervention—79.2%; control—81%	BIACA	Individual families. Parents may have been present in child-focused components dependent on child's clinical and developmental needs	16	16 weeks	3 months follow-up (CBT group only)	PARS, ADIS-CSR, MASC-P, RCMAS	Clinician, Parent, Child
White et al. [50]	Intervention: N = 13; control (wait list): N = 12	Overall sample—175 months; intervention—170 months; control—180 months	Overall sample—77%; intervention—73%; control—80%	MASSI	Individual and group	13 individual, 7 group	14 weeks	None reported	PARS, CASI-Anx	Clinician, parent
McConachie et al. [51]	Intervention: N = 17; control (delayed therapy): N = 14	Intervention—11.70 (1.40); control—11.80 (1.30)	Intervention—88%; control—87%	Exploring feelings	Group	7	Endpoint assessment undertaken 3 months after baseline	6 and 9 months after baseline (rate of change reported for parent and child measures)	ADIS, SCAS-P, SCAS-C	Clinician, parent, child
Storch et al. [52]	Intervention: N = 16; control (TAU): N = 15	Overall sample—12.74 (1.34); intervention—12.75 (1.24); control—12.73 (1.49)	Intervention—75%; control—86.7%	BIACA	Individual families	16	16 weeks	1 month follow-up (CBT group only)	PARS, ADIS-CSR, RCDAS, MASC-P	Clinician, child, parent
Wood et al. [26]	Intervention: N = 19; control (wait list): N = 14	Overall sample—12.30 (1.14); intervention—12.40 (1.30); control—12.20 (0.98)	Overall sample—70%; intervention—68%; control—71%	BIACA	Individual families (30 min individual sessions each and 30 min with parents and child together)	16	16 weeks	1 month follow-up (analysis outcomes between post and 1 month assessment reported)	ADIS-IV-C/P, MASC-P, PARS, RCADS	Clinician, parent, child
Clarke et al. [53]	Intervention: N = 14; control (wait list): N = 14	Intervention—12.64 (0.85); control—12.86 (0.70)	Overall sample—100%; intervention—100%; control—100%	Exploring feelings	Group	6	6 weeks	6–8 weeks post-intervention	SCAS-P, SCAS-C	Parent, child
Conaughton et al. [28]	Intervention: N = 20; control (wait list): N = 18	Overall sample—9.74 (1.30); intervention—9.81; control—9.67	Overall sample—85.7%; intervention—76.2%; control—95.2%	BRAVE-ONLINE program	Individual	10 child, 6 parent. Two booster sessions undertaken 1 and 3 months after completion of program	10–14 weeks	3 months follow-up (CBT group only)	SCAS-P, SCAS-C	Parent, child
Luxford et al. [54]	Intervention: N = 18; control (wait list): N = 17	Overall sample—13.20 (1.10)	Overall sample—89%	Exploring feelings	Group	6	3 months (6 weeks, 6 weeks follow-up)	6 week follow-up	SCAS-P, SCAS-C	Parent, child
Murphy et al. [55]	Intervention: N = 17; control (counselling): N = 19	Intervention—14.94 (1.63); control—15.56 (1.91)	Intervention—59%; control—63%	MASSI CBT program	Individual and group	12 individual (plus one booster if needed) and 5 group	Not reported	12 weeks follow-up	ADIS-C, CASI-anx	Clinician, parent
Cook et al. [31]	Intervention: N = 14; control (wait list): N = 17	Overall sample—5.45 (0.83); intervention—5.50 (0.88); control—5.42 (0.81)	Overall sample—87.1%; intervention—85.7%; control—88.2%	Fun with feelings program	Group	10	9 weeks + 1 booster session one month later	3 months follow-up (CBT group only)	CBCL-Anx	Parent
Maskey et al. [56]	Intervention: N = 14; control (wait list): N = 13	Overall sample—129.56 months (24.78); intervention—130.13 (28.38); control—129 (21.51)	Overall sample—78.1%; intervention—81.3%; control—75%	Simplified CBT technique and VR sessions	Individual	1 CBT, 4 VR	Not reported	Immediate treatment group one additional follow-up at 12 months (target behaviours only)	SCAS-P, SCAS-C	Parent, child
Wood et al. [42]	Intervention 1: N = 66; intervention 2: N = 59; control (TAU): N = 18	Not reported	BIACA—72%; coping CAT—82%; control—100%	(1) Standard of practice CBT (Coping Cat), (2) BIACA	Individual	16	16 weeks	6 month follow-up (data not reported in paper)	PARS, CBCL-Anx	Clinician, parent
Kilburn et al. [29]	Intervention: N = 25; control (wait list): N = 24	Overall sample—11.34 (1.77); intervention—11.99 (1.70); control—10.68 (1.60)	Overall sample—57%; intervention—60%; control—54%	Cool Kids ASD program	Group	10	13 weeks (10 weekly sessions with a 1-week break after sessions 3, 6, 8)	3 months (combined group follow-up assessment)	SCAS-P, SCAS-C	Parent, child

*ADIS* Anxiety Disorders Interview Schedule, *ADIS-C/P* Anxiety Disorders Interview Schedule for Children/Parents, *CASI-anx* Child and Adolescent Symptom Inventory-4, *CBCL-anx* The Child Behaviour Checklist anxiety subscale, *PARS* Paediatric Anxiety Rating Scale, *MASC* Multidimensional Anxiety Scale for Children, *RCDAS* Revised Child Anxiety and Depression Scales, *SCAS-P SCAS-C* Spence Children’s Anxiety Scale-Parent and Child versions

The number of CBT treatment sessions ranged between 5 and 20, with 16 sessions being the modal number—typically occurring on a weekly basis. Nine studies used group CBT, 8 used individual CBT and 2 had a combination of group and individual sessions (Additional file 2).

The majority of studies used an adapted version of CBT for autistic youth. Commonly used CBT interventions included: Behavioural Interventions for Anxiety in Children with Autism (BIACA) (k = 4), Exploring Feelings (k = 3), Coping Cat (k = 2) and Cool Kids (k = 2). A summary of our inclusion criteria and implemented CBT interventions for included studies is given within the Additional file 2.

The most commonly used parent and child report measures of anxiety were the Spence Children’s Anxiety Scale-Parent [57] and Child [58] versions (k = 9) and for clinicians, either the Anxiety Disorders Interview Schedule [59] (k = 8) or the Paediatric Anxiety Rating Scale [60] (k = 5). A range of other scales were also adopted notably for parent and child rated anxiety. We pooled and averaged effect sizes for studies where more than one measure was used by an informant.

### Parent rated

Nineteen studies (20 samples) compared CBT with a control group using parent-rated measures of anxiety. One study used two parent-rated measures [52] and so reported outcomes from the measures for these studies were combined. Additionally, two studies included two interventions compared to one control group [41, 42]—this was dealt with by halving the size of the control group and making comparisons with each intervention group separately.

**Fig. 2:**
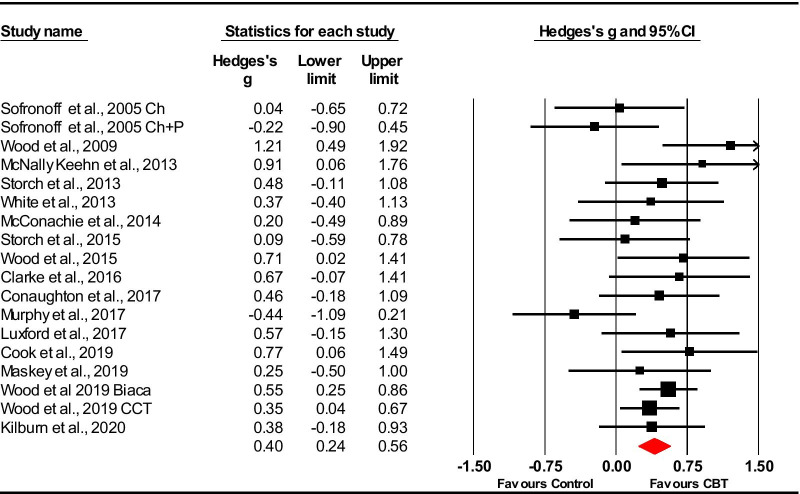
Forest plot of parent-rated measures of anxiety (minus Chalfant et al. [46])

Parent reported outcomes for anxiety in autistic youth showed CBT as an efficacious treatment in comparison to control conditions. Using a random effects model, the overall pooled effect size was medium (*g* = 0.55 [95% CI 0.26–0.84]). The studies were heterogeneous [*Q*(18) = 74.55, *p* < 0.001], with an *I*^*2*^ value of 75.86. However, one study [46] (*g* = 4.34) was an outlier showing no overlap of 95% CI with any other studies—this same study by Chalfant et al. was also excluded by previous meta-analyses [34, 36, 38]. Sensitivity analysis was used to explore the effect of this on the model by removing this outlier. Removing this study from the model reduced the overall Hedge’s *g* effect size to 0.40 [95% CI 0.24–0.56], showing that CBT remains efficacious at reducing anxiety compared with controls (see Fig. 2). Heterogeneity also greatly reduced [*Q* (17) = 22.03, *p* = 0.018], with an *I*^*2*^ value of 22.84.

Observation of funnel plots and the Trim and fill analysis suggested the possibility of one potentially missing trial reducing the effect size (*g* = 0.36, [95% CI 0.19–0.54]).

#### Follow-up

Nine trials (10 samples) contained follow-up data, but only 5 samples presented follow-up data for the controls [41, 53–55]. A random effects meta-analysis identified no significant reduction of anxiety at follow-up (*g* = 0.50 [95% CI − 0.10 to 1.12]). Heterogeneity was high [*Q(4)* = 14.8, *p* = 0.32; *I*^*2*^ = 72.98].

### Clinician rated

Ten studies (11 samples) compared CBT with a control group using clinician-rated measures. Three of the studies used two clinician-rated measures [26, 27, 52] and so effect sizes for reported outcomes from the measures for these studies were pooled.

**Fig. 3:**
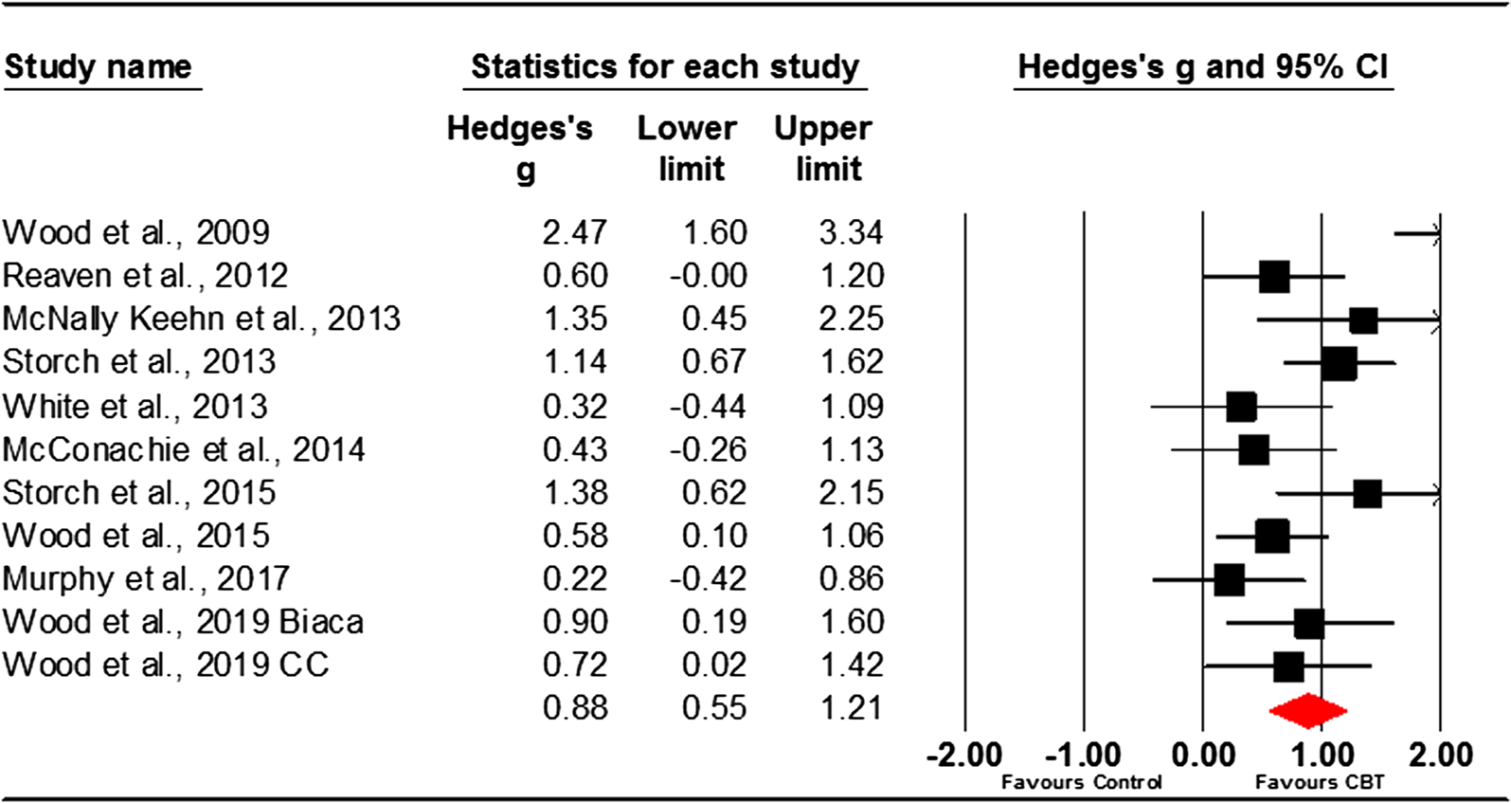
Forest plot of clinician-rated measures of anxiety

Clinician reported outcomes for anxiety in autistic youth showed CBT as an efficacious treatment in comparison to control conditions for reducing symptoms of anxiety. Using a random effects model, the overall pooled effect size was large, *g* = 0.88, 95% CI 0.55–1.21. The studies were heterogeneous, (*Q*(10) = 26.74, *p* < 0.001), with an *I*^*2*^ value of 62.61 (see Fig. 3).

Examination of funnel plots and Trim and Fill analysis revealed no evidence of publication bias.

#### Follow-up

Six trials provided follow up data, but only one had follow-up data for controls [55] so no analysis was conducted.

### Child/young person self rated

Thirteen studies included in the meta-analysis compared CBT with a control group using child-rated measures of anxiety. One of the studies used two child-rated measures [46] and so reported outcomes from the measures for these studies were combined.

**Fig. 4:**
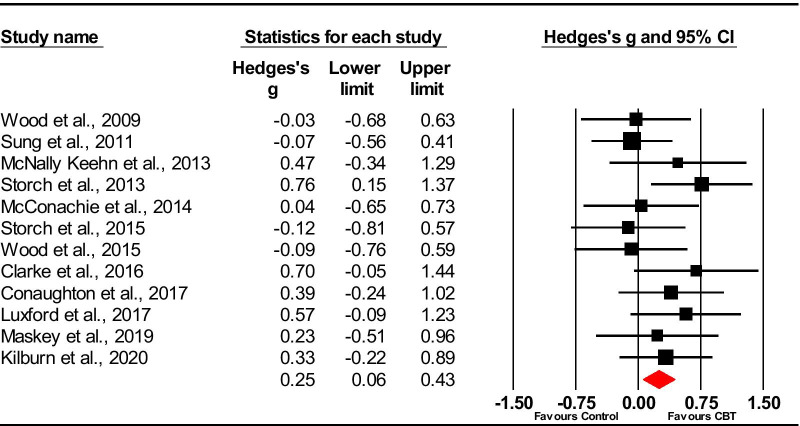
Forest plot of child-rated measures of anxiety (minus Chalfant et al. [46])

Using a random effects model, the overall pooled effect size was *g* = 0.47 (95% CI − 0.02 to 0.93). The studies were heterogeneous, (*Q(*12) = 78.77, *p* < 0.001), with an *I*^*2*^ value of 84.76. One study [46] (*g* = 2.93) was an extreme outlier owing to the much larger effect size, whose confidence intervals failed to overlap with any other included trials. Sensitivity analysis was used to explore the effect of this on the model by removing this outlier. Removing this study from the model reduced the heterogeneity [*Q*(11) = 10.43, *p* = 0.49], with an *I*^*2*^ = 0.00 and the effect size although it remained significant (*g *= 0.25 [95% CI 0.06–0.43]) (see Fig. 4). Inspection of funnel plots and trim and fill analysis revealed no missing studies.

#### Follow-up

Seven trials provided follow-up data, but only three provided follow-up data for controls [48, 53, 54]. A random effects meta-analysis on these trials indicated no significant benefit of CBT (*g* = 0.12, [95% CI − 0.23 to 0.47]). Heterogeneity was low (*Q*(2) = 0.67, *p* = 0.71; *I*^*2*^ = 0%).

### Moderator analyses

Four continuous variables (age, year of publication, proportion of male participants, number of sessions and proportion of participants with Asperger’s Syndrome) were examined as moderators using meta-regression analyses (see Table 2). All meta-regression analyses were conducted using a Method of Moments approach. These analyses revealed that the only significant moderator for effect sizes was age (for parent and clinician ratings), indicating that trials produced larger effect sizes in younger children. The failure to find any effect of number of sessions may well reflect the fact that the majority of trials have used 16 sessions. Similarly, the proportion of males per trial showed little variation, with most trials being male-dominated.

**Table 2:** Meta regression moderator analyses

	Parent (k = 17)	Clinician (k = 11)	Child (k = 12)
Age	Z = − 1.94, *p* = .05	Z = − 2.23, *p* = .025	Z = − 0.46, *p* = .66
Year of publication	Z = 0.71, *p* = .48	Z = − 1.79, *p* = .07	Z = 1.19, *p* = .23
Proportion of males	Z = 1.28, *p* = .20	Z = 0.21, *p* = .83	Z = 0.58, *p* = .56
Number of sessions	Z = 0.67, *p* = .50	Z = 0.41, *p* = .68	Z-1.26, *p* = .20
Proportion of asperger	Z = − 0.71, *p* = .47 (k = 16)^a^	Z = − 0.61, *p* = .54	Z = − 0.85, *p* = .40

^a^One trial (Cook et al. [31]) included AD and Aspergers but did not say how many of each

As effect sizes for clinicians, parents and child ratings differed substantially in the analyses outlined above, we performed a subgroup analysis comparing effect sizes in 6 trials where all three types of ratings were administered in the same groups of children [26, 27, 41, 47, 49, 50]. The pattern of effect sizes was comparable to the larger analyses, although this smaller analysis identified a much larger effect size for clinicians (1.71 [0.66–1.69]), slightly larger for parents (*g* = 0.58 [0.24–0.91]); however, self-ratings were non-significant and comparable to those obtained in the larger set (*g* = 0.26 [− 0.01 to 0.53]). Clinician ratings were significantly greater than child ratings (Q = 9.5 [*df* = 1], *p* = 0.002) and just failed to reach significance when compared to parental ratings (Q = 3.59 [*df* = 1], *p* = 0.06); while child and parental ratings did not significantly differ (Q = 2.12 [*df* = 1], *p* = 0.15).

We also used subgroup analysis to compare group versus individual format therapies for each rating informant. Clinician rating for group and individual formats (*g *= 0.53 [0.07–0.99], k = 2) versus *g* = 1.16 [0.74–1.58], k = 7) identified a significantly larger effects for individual therapy (Q = 5.58, *df* = 1, *p* = 0.04). Parent ratings for group versus individual formats (*g* = 0.33 [0.07–0.59]; k = 7) vs *g* = 0.50 [0.33–0.67]; k = 9) did not differ significantly (Q = 1.17, *df* = 1, *p* = 0.28); nor did child ratings for group versus individual (*g* = 0.32 [0.04–0.60]; k = 6) versus (*g *= 0.06 [.− 0.28 to 0.39]; k = 4) differ significantly (Q = 1.42, *df* = 1, *p* = 0.23).

Earlier trials, using different diagnostic criteria tended to also include a number of children who were given a diagnosis of Pervasive Developmental Disorder-Not Otherwise Specified (PDD-NOS): Wood et al. [47] (85%); Sung et al. [48] (83%); Reaven et al. [30] (6%); McNally et al. [49] (5%); Storch et al. [27] (41%); White et al. [50] (13%); Storch et al. [52] (40%); Wood et al. [26] (43%). Because PDD-NOS was diagnosed in a minority of trials, we conducted sub-group analyses comparing effect sizes in trials with PDD-NOS diagnoses (k = 8) vs those without (k = 11). This revealed no difference in effect sizes for ratings by clinicians (Q = 1.03, *p* = 0.31), parents (Q = 1.75, *p* = 0.19) or children themselves (Q = 1.32, *p* = 0.25).

We intended to analyse the categorical impact of blind versus non-blind outcome assessment. Of course, both parents and children/young people were aware of assignment and so non-blind. By contrast, all clinician ratings appeared to be blind at outcome and thus we could not analyse as a moderator within any rating type.

#### Risk of bias

All trials were evaluated for risk of bias using the Cochrane Risk of Bias Tool (RoB2) [38] by two authors (DG & KL). Of the 19 trials, 4/19 (21%) were at low risk of bias, 12/19 (63%) some concerns and 3/19 (16%) were at high risk of bias. The main area of concern was ‘reporting bias’, with only 7/19 (37%) registering their protocol (see Table 3).

**Table 3:** Risk of bias for included studies

Study (date)	Selection bias	Performance bias	Attrition bias	Detection bias	Reporting bias	Overall bias
Sofronoff et al. [41]	Low	Low	Low	High	Some concerns	High
Chalfant et al. [46]	Low	Low	High	High	Some concerns	Some concerns
Wood et al. [47]	Low	Low	Low	High	Some concerns	Some concerns
Sung et al. [48]	Low	Low	Low	Low	Some concerns	Some concerns
Reavan et al. [30]	Low	Low	Low	Low	Some concerns	Some concerns
McNally Keehn et al. [49]	Low	Low	Low	Low	Some concerns	Some concerns
Storch et al. [27]	Low	Low	Low	Low	Some concerns	Some concerns
White et al. [50]	Some concern	Low	Low	Low	Some concerns	Some concerns
McConachie et al. [51]	Low	Low	Low	Low	Low	Low
Storch et al. [52]	Low	Low	Low	Low	Low	Low
Wood et al. [26]	Low	Low	Low	Low	Low	Low
Clarke et al. [53]	Low	Low	Low	Low	Some concerns	Some concerns
Conaughton et al. [28]	Low	Low	Low	Low	Some concerns	Some concerns
Luxford et al. [54]	Low	Low	Low	High	Some concerns	High
Murphy et al. [55]	Low	Low	Low	Low	Some concerns	Some concerns
Cook et al. [31]	Low	Low	Low	High	Low	High
Maskey et al. [56]	Low	Low	Some concerns	Low	Some concerns	Some concerns
Wood et al. [42]	Low	Low	Low	Low	Some concerns	Some concerns
Kilburn et al. [29]	Low	Low	Low	Low	Low	Low

## Discussion

This systematic review and meta-analysis aimed to assess the evidence base on the efficacy of CBT in reducing anxiety in children and young people on the autism spectrum. We included data derived only from RCTs and considered treatment efficacy from the perspective of multiple informants including parent, clinician and child/ self-report. Importantly, we also summarised what is known about the durability of treatment gains. This has been missing in previous evidence syntheses in this area [34, 36, 38].

Nineteen studies were included with data from 833 participants. The most common modality of intervention delivery across trials was group (n = 9) or individual therapy (n = 8), with two trials using a combination of these approaches. Some degree of consistency was apparent in the duration of intervention across trials, with the modal number being 16 sessions. All studies reported the use of CBT protocols that had been adapted for use with autistic youth. Most included parent and or clinician ratings of anxiety, with 13 also including self-rating by the child/young person. Interestingly, a wide range of assessments were used to profile symptoms of anxiety, but not all have support for their use in ASD trials [61, 62] or at least not without modifications to allow more robust interpretation. Building more consensus on the psychometric soundness and necessary adaptions to measure anxiety as an endpoint in ASD will be important as more trials emerge. Notably, none of the trials included health economic evaluation, despite this forming an important framework for assessing value in health interventions [63].

The current meta-analyses confirm the efficacy of CBT in reducing anxiety in autistic youth, and thus concurs with previous reviews [34, 36, 38], and clearly establishes that effect sizes vary markedly across informants. For clinician ratings, which were blind, we found a large effect (*g* = 0.88) versus a moderate effect for parent rated symptoms (*g* = 0.58), although the latter reduced following the removal of one extreme outlier (*g* = 0.42). For child/self-rated symptoms, we found a small to moderate effect (*g* = 0.47), which again reduced after removal of one extreme outlier (*g* = 0.25). A direct analysis in 6 trials using all three informant ratings confirmed a larger effect size for clinicians (*g =*1.71 [0.66–1.69]) than for both parents (*g *= 0.58 [0.24–0.91]) and the self-ratings of children, with the latter being non-significant (*g *= 0.26 [− 0.01 to 0.53]) in this sub-analysis.

Turning to factors that might moderate the efficacy of CBT, we could divide them broadly into those relating to the participants, and those relating to the therapy itself. Turning to participant variables, meta-regression suggested that the benefits of CBT may be more pronounced in younger children. This may suggest better developmental tailoring of intervention protocols, with room to enhance therapeutic techniques for older children. Gender was not a significant moderator, although females are generally under-represented in autism research, impacting the power of an analysis to detect a true effect. We also found no effect for the proportion of those identified as having Asperger syndrome or PDD-NOS. Turning to effects related to the therapy itself, the duration of CBT was not found to be a contributing factor, but this likely reflects most trials including a similar number of sessions (i.e. 16). Our subgroup comparison of group versus individual CBT revealed only one significant advantage for individual therapy for clinician ratings; all other informant rating revealed no differences. The advantage for individual therapy for clinician ratings however needs to be interpreted cautiously as it involved just 2 group versus 7 individual trials.

An important difference between the current meta-analysis and those of both Ung et al. [36] and Perihan et al. [34] concerns the fact that the latter both pooled data across informant. As with the earlier meta-analysis by Sukhodolsky et al. [38], we calculated separate effect sizes for different informants (clinician, parent and child). In the current larger sample of RCTs (more than twice as many), we similarly found large variability across informant ratings—with clinicians producing a large effect, a moderate effect for parents and a small effect for the children/ young people themselves. Furthermore, this informant discrepancy pattern emerged in studies using all three informant measures, and so, does not reflect inter-trial variability. Informant discrepancies have been acknowledged as ubiquitous in research on the assessment, development, and treatment of childhood problems [64]. Indeed, a meta-analysis of 341 studies published between 1989 and 2014 reported low-to-moderate correspondence for cross-informant estimates [65]. Rather than being averaged away, informant inconsistency requires careful investigation.

The most notable feature of course is the large effect size reported for clinicians and the fact that the children/young people themselves report little or no reduction of anxiety following CBT. The clinician and self-ratings reported here also differ in one other critical manner and that is that all clinician ratings were made blind. Arguably, blinded clinician ratings reporting greater symptom improvement might reflect the ability of skilled professionals to unpick the overlap of anxiety symptoms from features of autism per se, though this distinction is a source of debate [66]. Our finding of a much larger effect size for blinded outcome assessment runs counter to the more typical finding that non-blind assessment inflates effect sizes [67] and further raises important questions about how we assess and who assesses outcome in this group and what constitutes a meaningful gain. Given that the children/young people themselves are reporting little change in their anxiety, it might be tempting to think that either they are reluctant or somehow unable to identify their anxiety and that the adult ratings are more accurate. Informant discrepancies are likely to be greater for internalising (such as anxiety and mood) over externalising mental health concerns (such as aggression and hyperactivity) [68]. However, the difference between the two sets of adult raters (clinicians and parents) was just as large; and it does seem as though clinicians are producing much greater effect sizes than either parents or children.

The lack of consensus on the ‘gold standard’ measure of anxiety for autistic youth [61] adds nuances in intervention research where different assessment tools are used across studies and vary in relevance. For example, the Spence Children’s Anxiety Scale-Parent Version (SCAS-P) [57] was used in several studies within our review. It has been suggested that whilst this scale is useful, it requires adaptation when applied to children on the autism spectrum. Using the SCAS-P in its original form has been questioned [62, 69], for example, owing to perceived non-applicability of items and the psychometric structure of the scale differing. Similarly, for self-rated-symptoms, engagement requires items to be relevant and the informant to have the degree of language and cognitive skills to accurately complete [61]. The correlation between parent and child ratings on the Spence Children’s Anxiety Scale are favourable for children in the general population, whereas ASD parent–child reports are typically poorly correlated [70]. Of course, it may be that measures developed for youth in the general population have comparable sensitivity, specificity, and factor structure when employed in autistic youth [71] and discrepancies reflect real differences. There are two ways to explore these assertions further. The first is for future trials to utilise measures of parent and self-report anxiety that are developed specifically for autistic youth. Such measures should offer a more relevant and reliable profile of symptoms by informants. For example, the 25-item Parent Reported Anxiety Scale (PRAS-ASD) [72] or the anxiety scale for children with autism spectrum disorder (ASC-ASD) [73]. We also advocate qualitative follow-up in trials to uncover how youth and parents describe intervention benefits. This would further insights into mechanistic elements, and crucially, offer explanations as to why differences exist between the informants of anxious symptoms.

Where outcome reporting beyond post-treatment effects was included in trials, we restricted our analyses to studies that did this consistently (i.e. control participants included). Although we identified 23 sets of follow-up data from various informant groups, only 8 followed-up the control group and these all came from 5 trials. This resulted in synthesis of parent and child rated anxiety only and yielded no support for sustained treatment effects at follow-up (range 1–9 months). Whilst data from studies in the general population have supported the long-term effectiveness of CBT for anxiety in youth [74], we might expect different end states for youth on the autism spectrum where other daily-challenges could interfere with ratings of overall quality of life [61]. Given the small numbers of trials with follow-up data, we would apply some caution in interpreting. Nonetheless, parents, providers and service commissioners in particular will benefit from knowing the extent to which CBT facilitates lasting improvement in functioning; and such information will help set expectations for families. By identifying predictors of sustained gain versus relapse, clinicians might also be able to better optimise treatment protocols and so future trials need to attend to this.

Some caveats need to be acknowledged when interpreting the current findings. The first relates to the assessment of risk of bias, which had not been examined in the three previous meta-analyses [34, 36, 38]. Only three trials were at low risk of bias, with the majority having ‘some concern’ and three studies were rated as at high risk of bias, mostly due to ‘reporting bias’. As the field of clinical trials advances further, the requirement to prospectively register study protocols should address the lack of clarity around whether trials have selectively published outcomes. Reporting bias is under-recognised and impacts on the interpretation of findings from clinical trials [75]. Second, the large amount of heterogeneity in studies including clinician ratings could not be explained by the moderator analyses undertaken. This has made it difficult to explain treatment effects with available information about study and participant characteristics. The added detection of publication bias in these studies should also be viewed with caution. Third, we are unable to make any definitive conclusions about sustained improvement in symptoms beyond posttreatment effects because of the small numbers of studies analysed. It will be important for future trials to include this data so that durability of intervention gains can be fully explored. Most studies included in this meta-analysis also had small samples (the mean receiving CBT was 23 and controls 18) and were underpowered to detect even the largest effect size reported for clinicians. The current results suggest that the optimal number will vary depending upon which informant ratings are taken from. Finally, we acknowledge that our own review was not pre-registered, though all data have been made available with details of the search results itself (i.e. Fig. 1).

## Conclusions

This study provides the largest meta-analysis (19 RCTs and 833 participants) to date on the efficacy of CBT for anxiety in children and adolescents on the autism spectrum. Our primary findings concur with previous reviews showing that CBT reduces anxiety in the immediate posttreatment period. Indeed, ratings by all three informant types were consistent in documenting a significant reduction of anxiety; and showed little or no evidence of publication bias. Nevertheless, the substantial variability in effect sizes across clinicians, parents and children would advise against future meta-analyses pooling across informants. It is notable that risk of bias was low except in the domain of reporting bias, where risk was generally higher. The latter seems to reflect the lack of preregistration of trials.

Recommendations for future trials include a focus on selecting autism-specific measures of anxiety or at least making necessary adaptions to scales; prospective registration of trials to overcome potential reporting biases; and the inclusion of follow-up analyses to assess whether treatment gains are sustained. We also note the absence of reporting on adverse events and the relative absence of health economic evaluation across trials, both of which are crucial to determine the wider value of the intervention beyond the reduction of symptomatology.

## Supplementary Information

**Additional file 1**. PRISMA Checklist.

**Additional file 2**. Additional study information on included studies.

## Data Availability

For queries about specific papers used within the review, please contact the corresponding author of the relevant study. All data used with the systematic review and meta-analyses are available within the supplementary files.

## Abbreviations

ASD: Autism spectrum disorder
DSM: Diagnostic and Statistical Manual of Mental Disorders
ICD: International Classification of Diseases
CBT: Cognitive behavioural therapy
RCT: Randomised Controlled Trial
PRISMA: Preferred Reporting Items for Systematic Reviews and Meta-Analyses
RoB2: Revised Cochrane risk of bias tool for randomised trials
SCAS-P/c: Spence Children’s Anxiety Scale-Parent/Child
ADIS: Anxiety Disorders Interview Schedule
PARS: Paediatric Anxiety Rating Scale
MASC: Multidimensional anxiety scale for children
CBCL- Anx: The Child Behaviour Checklist anxiety subscale
ADIS-C/P: Anxiety Disorders Interview Schedule for Children/Parents
CASI-anx: Child and Adolescent Symptom Inventory-4
RCDAS: Revised Child Anxiety and Depression Scales
PRAS- ASD: Parent reported anxiety
ASC-ASD: Anxiety Scale for Children with Autism Spectrum Disorder
