# Supplementary material for: Cognitive behavioural therapy for anxiety in children and young people on the autism spectrum: a systematic review and meta-analysis
Source: BMC Psychol. 2021 Oct 1;9:151. doi: 10.1186/s40359-021-00658-8 (PMC8487131; doi:10.1186/s40359-021-00658-8)
Supplement: Supplementary file 2 — Additional file 2. Additional study information on included studies. [file 40359_2021_658_MOESM2_ESM.docx]

**Supplementary Table 1: Additional study information on included studies**

| **Study Authors** | **Inclusion criteria** | **CBT Type** | **CBT intervention summary** |
| --- | --- | --- | --- |
| **Sofronoff et al., 2005^42^** | a) aged 10-12 years, b) primary diagnosis of Asperger’s syndrome from paediatrician, c) semi-structured phone interview with parent. | CBT | Designed to be highly structured, entertaining and informative. Use of workbook and materials for each session including information on being happy, relaxed and anxious, with space for comments and question responses. Project to complete after each session. Sessions explored: 1) happiness and relaxation, 2) anxiety and bodily changes, 3) social tools and thinking tools, 4) measures of the degrees of emotion, 5) social stories for emotion management, 6) designing a programme to improve anxiety management. |
| **Chalfant et al., 2007**^47^ | a) diagnosis of ASD, b) age appropriate language skills, c) primary anxiety disorder diagnosis on basis of structured clinical interview with parent and child using ADIS, d) met criteria for an anxiety disorder beyond their ASD related symptoms. | Cool Kids program | Designed as a high functioning autism adaptation to the Cool Kids program (Macquarie University). Focuses on treating the main components of anxiety. The adapted material covered the recognition of anxious feelings and somatic reactions to anxiety, simplified cognitive restructuring activities, coping self-talk, exposure to feared stimuli and relapse prevention. The first 4 sessions were training sessions and introduced role-playing anxiety management procedures, and the remaining sessions were practice sessions consolidating newly learned skills and planning out weekly exposure tasks. Adaptations were made to account for learning styles of children with high functioning autism. The program was extended over a longer period of time (6 months), used more visual aids and structured worksheets and the largest components of the program focused on relaxation and exposure. Information in cognitive activities was simplified. |
| **Wood et al., 2009**^48^ | Children were referred to the study. Inclusion: a) met research criteria for diagnosis of autism, Asperger’s, or PDD-NOS, b) met research criteria for at least one anxiety disorder (SAD, social phobia or OCD), c) were not taking psychiatric medication at baseline or taking a stable dose prior to baseline, d) if used, maintained same dosage throughout study, e) verbal IQ >70. | Building confidence CBT program | Program was modified for use with children with ASD. The manual includes coping skills and training followed by in vivo exposure. Hierarchy created in which feared situations are ordered from least to most distressing. Children work their way up the hierarchy, and are rewarded as they attempt more fearful activities. ASD specific treatment modules included teaching parents and children friendship skills, and children receive social coaching. Skills are practiced at home, school, play dates etc. and are reinforced via a reward system. Children’s circumscribed interests and stereotypies incorporated into the intervention in two ways: to build rapport and later via a suppression approach. |
| **Sung et al., 2011**^49^ | a) 9-16 years, b) clinical diagnosis of Autism, Asperger syndrome, PDD(NOS) or ASD by DSM-IV criteria, c) classification of Autism or Autism Spectrum on the ADOS, d) verbal comprehension of 80 and above and perceptual reasoning skills of 90 and above on WISC-IV, e) for children on medication, no change of dosage 1 month prior to start of study and throughout study duration. | Modiﬁcations and adaptations from various CBT programs, including Coping Cat program, Exploring feelings and unpublished anxiety management programs from the CGC and Autism Resource Centre. | Developed by Psychologists from the Child Guidance Clinic and the Autism Resource Centre (Singapore). Incorporated strategies found to be effective with children with ASD, such as visual strategies, structure, role-play and social stories. Program tailored to meet cultural and developmental needs of children with ASD in the Asian population. Scenario examples and worksheets also tailored to context more meaningful to children in Asia. CBT program comprised 3 main components: 1) recognition and understanding of emotions, 2) anxiety management techniques, 3) problem-solving strategies based on the STAR strategy. |
| **Reaven et al., 2012**^31^ | a) Aged 7-14 years, b) confirmed diagnosis of an ASD by clinical psychologist, c) speaking in full complex sentences, d) clinically significant symptoms of anxiety. | Facing your fears (FYF) | Multi-family group sessions supported by a set of manuals for facilitator, parents and youth. Developed specifically for children with ASD. Incorporated important components from previously supported programs (e.g. Coping Cat) while making appropriate adaptations for children with high functioning ASD. |
| **McNally Keehn et al., 2013**^50^ | a) Diagnosis of ASD based on the ADOS, ADI-R and expert clinical judgement based on DSM-IV criteria, b) diagnosis of SAD, SP or GAD made on the basis of the ADIS-P, c) Full-Scale IQ ≥ 70 confirmed by WASI, d) age 7-14 years, e) English as primary language. | Coping Cat program | Designed for children and young adolescents with SAD, SP and GAD. Primary goal is to teach children to recognise signs of anxious arousal and to let these signs serve as a cue to implement anxiety management techniques. Sessions 1-8: skills training, Sessions 9-16: exposure tasks in a hierarchical sequence of anxiety-provoking situations. Two parent only sessions (at sessions 4 & 9). Modifications were made to accommodate children's learning styles and improve treatment success. |
| **Storch et al., 2013**^28^ | Children aged between 7-11years. Inclusion: a) diagnosis of autistic disorder, Asperger’s, or pervasive developmental disorder–not otherwise specified, b) or primary diagnosis of SAD, social phobia, GAD, OCD. Primary diagnosis determined through ADIS-C/P, minimum score of 14 on PARS. | BIACA | Based on a CBT manual for typically developing anxious youths. Uses a modular treatment approach. Incorporates problematic anxiety and non–anxiety-based symptoms as treatment goals. Considers barriers to working with children with ASD. Family based approach - one module completed by child and one by parent. Core of intervention consistent with CBT for typically developing children (at least 3 sessions on coping skills and 8 on in vivo exposures to feared stimuli), treatment followed a modular, flexible format with therapy modules chosen on an ongoing basis. Additional 5 sessions covered modules based on child's current clinical need. |
| **White et al., 2013**^51^ | a) aged 12-17 years, b) diagnosis of ASD supported by ADOS and ADI-R, c) met diagnostic criteria for at least one of 4 anxiety disorders, SoP, GAD, SP, or SAD, determined by ADIS-C/P, d) verbal IQ of 70 or above and no previous diagnosis of intellectual disability. | MASSI | Manual based treatment program delivered via: individual therapy (up to 13 sessions), group therapy (skills practice, 7 sessions), parent education and coaching (after each individual therapy session). Treatment specific case was developed after the third individual session and treatment proceeded to address participants specific anxiety symptoms and social skill deficits. Appropriate treatment modules were selected form the MASSI manual by the therapist for the individual sessions based on anxiety symptoms and social difficulties. Content of the group sessions was the same for all participants, as the skills covered broadly applicable for all adolescents with ASD. |
| **McConachie et al., 2014**^52^ | a) confirmed diagnosis of ASD, b) met criteria for at least one anxiety disorder, c) aged between 9-13 years 11 months with IQ > 69. Parents also willing to attend group therapy in parallel with child. | Exploring Feelings | Minor adjustments made to exploring feelings program for use. Introductory session added. Program activities designed to be highly structured and interesting. Sessions focused on: 1) how to identify feelings of being happy, relaxed or anxious, then 2) building a toolbox of strategies. "Project work" issued at end of each session to complete. |
| **Storch et al., 2015**^53^ | a) diagnosis of autism, Asperger’s, or PDD-NOS, b) co-occurring anxiety diagnosis of SAD, GAD, OCD or social phobia, c) score of >13 on the PARS, d) an IQ >80 on the Wechsler Abbreviated Scale of Intelligence or review of standardized testing in the previous 2 years. | BIACA | CBT sessions were according to developmentally modified version of the BIACA treatment manual that was used in Wood et al., (2015). However, in this study parents were included in the majority of the sessions (during child and parent oriented sessions) to address low treatment motivation, facilitate treatment progress and generalisation, promote autonomy, communication skills and problem solve treatment barriers. Treatment was concluded with a termination module addressing relapse prevention and continuing treatment progress. |
| **Wood et al., 2015**^27^ | a) diagnosis of ASD and at least one anxiety disorder with clinical severity level above 3, b) aged 11-15 years, c) IQ score of 85 or above on WISC-IV. | BIACA | Adaptations to traditional youth CBT programs to optimise treatment effectiveness. Sessions provided in modular format, selected on session by session basis to address clinical needs. Minimum 3 sessions on basic coping skills and 8 on in vivo exposure. A series of modules focused on core concerns of anxious adolescents who have autism. BIACA program emphasises key skills for positive social behaviour skills and developmentally appropriate get-togethers that can be used in real world settings. Parent sessions encouraged youth independence, increasing communication skills, and creating skills to diffuse problematic situations. Supporting home-based exposures and use of reward systems was highlighted. |
| **Clarke et al., 2016**^54^ | Secondary schools invited to participate. Children identified to take part based on schools concern rather than formal diagnosis. Requisite: a) for all children to have a multidisciplinary assessment of autism, with validated reports of diagnosis conforming to either DSM IV or ICD X. | Exploring feelings | Designed specifically for children with autism. Consists of aspects which are considered good practice for children with autism, such as visually presented material and comic strips conversations. Sessions explored: Strengths and special talents, feeling/being relaxed, bodily state when anxious, coping, relaxation techniques, understanding anxious situations etc. |
| **Conaughton et al., 2017**^29^ | a) Aged between 8-12 years, b) ability to read and write English at min. age 8 years level, c) access to a computer with internet access from home, d) min. diagnostic severity rating of 4 (based on 8-point clinician scale), comorbidity with other anxiety and externalising disorders permissible. | BRAVE-ONLINE program | Adapted from a clinic based CBT anxiety program for children aged 7-14 years. Based on research relating to psychosocial determinants of child anxiety and evidence based CBT interventions for child anxiety. Program covers various anxiety management strategies, and participants consolidate learning of these through completing weekly homework tasks. Online delivery includes a mid-program phone call and brief weekly email contacts with the internet therapist. |
| **Luxford et al., 2017**^55^ | Recruited from four mainstream secondary schools. Inclusion: a) formal diagnosis of ASD from a qualified health professional, b) verbal and total IQ score of >70, c) currently experiencing clinically significant symptoms of anxiety, measured by elevated scores for either teacher reported school anxiety (score >17) or parent reported anxiety (score >24 on SCAS-P). | Exploring Feelings | Uses developmentally appropriate language and materials designed for use with children with ASD. Active targeting of generalisation skills outside of the CBT session, in a naturalistic environment. Home project after each session. Participants work to create a "tool box" of anxiety strategies across sessions. |
| **Murphy et al., 2017**^56^ | Participants were young people and their parents attending 3 CAMHS clinics. Those invited to participate were: a) aged 12–18 years referred to the clinic between April 2011 and April 2013 with a diagnosis of ASD and anxiety, b) met diagnoses based on the ADOS, ADI-R and ADIS, c) IQ > 70. | MASSI CBT program | Developed as a treatment for young people with ASD and anxiety. Includes CBT for anxiety reduction and strategies targeting social skill deficits. Aimed to build rapport and encourage expression of feelings. Dealt with anxiety as and when raised, but with no focus on physical symptoms or cognitions. |
| **Cook et al., 2019**^32^ | a) aged 4-6 years, b) diagnosis of Asperger’s disorder, autistic disorder or autism spectrum disorder, c) experiencing clinically significant level of anxiety (total anxiety score > 48 on PAS). | Fun with feelings program | Parent mediated and specifically targeted anxiety in children aged 4-6 years with high functioning ASD and comorbid anxiety. Designed to help children and parents recognise anxious feelings and physiological reactions to anxiety, practice CBT based coping strategies to regulate emotions. Parents instructed in CBT strategies which they then taught to their children. |
| **Maskey et al., 2019**^57^ | a) Age 8-14 years, b) diagnosis of ASD, c) verbally fluent and able to understand instructions for treatment participation, d) completion of outcome measures. | Simplified CBT technique and VR sessions | Simplified CBT techniques. CBT elements repeated and consolidated during VR sessions. CBT techniques included: 1) identifying feelings; 2) visual ‘feeling thermometer’ using the participant’s words to describe anxiety; 3) two relaxation exercises; 4) identification of the participant’s positive coping statement to use in the treatment sessions. Goal for end of treatment was agreed with the participant and used for confidence rating charts. |
| **Wood et al., 2019**^43^ | a) age 7-13 years, b) having a clinical diagnosis of ASD confirmed by the study's clinical research evaluation, c) IQ of 70 or more points, d) anxiety (as defined by PARS total score of ≥14points) | 1) Standard of practice CBT (Coping Cat), 2) BIACA | Standard of practice CBT (Coping Cat) - Main features recognizing anxious feelings and somatic reactions to anxiety, identifying thoughts in anxiety provoking situations, developing a coping plan, in vivo exposure tasks, self-reinforcement for effort. Home-work tasks. Parent involvement: 15 min check-ins at start of each session, 2 meetings with therapist. BIACA - 90-minute sessions (split evenly between children and parents). Modular format using an algorithm to personalize treatment. Children’s disruptive behaviour is addressed as needed. Children taught social skills to facilitate peer based assignments. Children's special interests treated as an asset and incorporated into treatment to promote engagement. Target behaviours reinforced using reward system at home and in school. |
| **Kilburn et al., 2020**^30^ | a) official ASD diagnosis, b) anxiety symptoms clinically deemed severe enough to need independent treatment (clinically deemed), c) IQ >70 and age adequate language ability, d) no symptoms of active psychosis or ADHD. | Cool Kids ASD program | Specifically developed for children with an anxiety disorder comorbid with ASD. Adaptations included more visual aids, structured worksheets, simplified cognitive restructuring exercises, relaxation techniques, and concrete exposure tasks. |

**Supplementary Table 2: PRISMA checklist**

| **Section and Topic** | **Item #** | **Checklist item** | **Location where item is reported** |
| --- | --- | --- | --- |
| **TITLE** | | |  |
| Title | 1 | Identify the report as a systematic review. | 1 |
| **ABSTRACT** | | |  |
| Abstract | 2 | See the PRISMA 2020 for Abstracts checklist. | 2 |
| **INTRODUCTION** | | |  |
| Rationale | 3 | Describe the rationale for the review in the context of existing knowledge. | 5-7 |
| Objectives | 4 | Provide an explicit statement of the objective(s) or question(s) the review addresses. | 7 |
| **METHODS** | | |  |
| Eligibility criteria | 5 | Specify the inclusion and exclusion criteria for the review and how studies were grouped for the syntheses. | 8 |
| Information sources | 6 | Specify all databases, registers, websites, organisations, reference lists and other sources searched or consulted to identify studies. Specify the date when each source was last searched or consulted. | 8 |
| Search strategy | 7 | Present the full search strategies for all databases, registers and websites, including any filters and limits used. | 8 |
| Selection process | 8 | Specify the methods used to decide whether a study met the inclusion criteria of the review, including how many reviewers screened each record and each report retrieved, whether they worked independently, and if applicable, details of automation tools used in the process. | 8 |
| Data collection process | 9 | Specify the methods used to collect data from reports, including how many reviewers collected data from each report, whether they worked independently, any processes for obtaining or confirming data from study investigators, and if applicable, details of automation tools used in the process. | 9 |
| Data items | 10a | List and define all outcomes for which data were sought. Specify whether all results that were compatible with each outcome domain in each study were sought (e.g. for all measures, time points, analyses), and if not, the methods used to decide which results to collect. | See table 1 |
|  | 10b | List and define all other variables for which data were sought (e.g. participant and intervention characteristics, funding sources). Describe any assumptions made about any missing or unclear information. | See table 1 |
| Study risk of bias assessment | 11 | Specify the methods used to assess risk of bias in the included studies, including details of the tool(s) used, how many reviewers assessed each study and whether they worked independently, and if applicable, details of automation tools used in the process. | 10 |
| Effect measures | 12 | Specify for each outcome the effect measure(s) (e.g. risk ratio, mean difference) used in the synthesis or presentation of results. | 9 |
| Synthesis methods | 13a | Describe the processes used to decide which studies were eligible for each synthesis (e.g. tabulating the study intervention characteristics and comparing against the planned groups for each synthesis (item #5)). | See figure 1 |
|  | 13b | Describe any methods required to prepare the data for presentation or synthesis, such as handling of missing summary statistics, or data conversions. |  |
|  | 13c | Describe any methods used to tabulate or visually display results of individual studies and syntheses. | Figures 2,3, 4 |
|  | 13d | Describe any methods used to synthesize results and provide a rationale for the choice(s). If meta-analysis was performed, describe the model(s), method(s) to identify the presence and extent of statistical heterogeneity, and software package(s) used. | 10 |
|  | 13e | Describe any methods used to explore possible causes of heterogeneity among study results (e.g. subgroup analysis, meta-regression). | 10 |
|  | 13f | Describe any sensitivity analyses conducted to assess robustness of the synthesized results. | 10 |
| Reporting bias assessment | 14 | Describe any methods used to assess risk of bias due to missing results in a synthesis (arising from reporting biases). | 9 |
| Certainty assessment | 15 | Describe any methods used to assess certainty (or confidence) in the body of evidence for an outcome. | n/a |
| **RESULTS** | | |  |
| Study selection | 16a | Describe the results of the search and selection process, from the number of records identified in the search to the number of studies included in the review, ideally using a flow diagram. | See fig 1 |
|  | 16b | Cite studies that might appear to meet the inclusion criteria, but which were excluded, and explain why they were excluded. | See table 1 |
| Study characteristics | 17 | Cite each included study and present its characteristics. | See table 1 |
| Risk of bias in studies | 18 | Present assessments of risk of bias for each included study. | See table 3 |
| Results of individual studies | 19 | For all outcomes, present, for each study: (a) summary statistics for each group (where appropriate) and (b) an effect estimate and its precision (e.g. confidence/credible interval), ideally using structured tables or plots. | 12-17 |
| Results of syntheses | 20a | For each synthesis, briefly summarise the characteristics and risk of bias among contributing studies. | 20 |
|  | 20b | Present results of all statistical syntheses conducted. If meta-analysis was done, present for each the summary estimate and its precision (e.g. confidence/credible interval) and measures of statistical heterogeneity. If comparing groups, describe the direction of the effect. | 17-19 |
|  | 20c | Present results of all investigations of possible causes of heterogeneity among study results. | 17-19 |
|  | 20d | Present results of all sensitivity analyses conducted to assess the robustness of the synthesized results. | n/a |
| Reporting biases | 21 | Present assessments of risk of bias due to missing results (arising from reporting biases) for each synthesis assessed. | See appendix |
| Certainty of evidence | 22 | Present assessments of certainty (or confidence) in the body of evidence for each outcome assessed. | n/a |
| **DISCUSSION** | | |  |
| Discussion | 23a | Provide a general interpretation of the results in the context of other evidence. | 21-26 |
|  | 23b | Discuss any limitations of the evidence included in the review. | 26-27 |
|  | 23c | Discuss any limitations of the review processes used. | 27 |
|  | 23d | Discuss implications of the results for practice, policy, and future research. | 27-28 |
| **OTHER INFORMATION** | | |  |
| Registration and protocol | 24a | Provide registration information for the review, including register name and registration number, or state that the review was not registered. | n/a |
|  | 24b | Indicate where the review protocol can be accessed, or state that a protocol was not prepared. | 27 |
|  | 24c | Describe and explain any amendments to information provided at registration or in the protocol. | n/a |
| Support | 25 | Describe sources of financial or non-financial support for the review, and the role of the funders or sponsors in the review. | 29 |
| Competing interests | 26 | Declare any competing interests of review authors. | 29 |
| Availability of data, code and other materials | 27 | Report which of the following are publicly available and where they can be found: template data collection forms; data extracted from included studies; data used for all analyses; analytic code; any other materials used in the review. | See appendix |

**Supplementary File 3: Funnel plots for parent, clinician, and child anxiety assessments**

**Parent**

**Clinician**

**Child**
